# Exogenous Jaagsiekte Sheep Retrovirus type 2 (exJSRV2) related to ovine pulmonary adenocarcinoma (OPA) in Romania: prevalence, anatomical forms, pathological description, immunophenotyping and virus identification

**DOI:** 10.1186/s12917-020-02521-1

**Published:** 2020-08-17

**Authors:** Corina Toma, Valentin Adrian Bâlteanu, Septiumiu Tripon, Adrian Trifa, Alexandra Rema, Irina Amorim, Raluca Maria Pop, Roxana Popa, Cornel Catoi, Marian Taulescu

**Affiliations:** 1 Department of Veterinary Pathology, University of Agricultural Sciences and Veterinary Medicine, 3-5 Calea Manastur, 400372 Cluj-Napoca, Romania; 2 Laboratory of Genomics, Biodiversity, Animal Breeding and Molecular Pathology, Institute of Life Sciences, University of Agricultural Sciences and Veterinary Medicine, Cluj-Napoca, Romania; 3 National Institute for Research and Development of Isotopic and Molecular Technology, “C. Crăciun” Electron Microscopy Laboratory, Babeș-Bolyai University, Cluj-Napoca, Romania; 4 Department of Genetics, “Iuliu Haţieganu” University of Medicine and Pharmacy, Cluj-Napoca, Romania; 5 Department of Genetics, “Ion Chiricuta” Cancer Institute, Cluj-Napoca, Romania; 6 Institute of Biomedical Sciences Abel Salazar (ICBAS), University of Porto, Rua Jorge Viterbo Ferreira nr.228, 4050-313 Porto, Portugal; 7 Department of Pharmacology, Toxicology and Clinical Pharmacology, Iuliu Haţieganu University of Medicine and Pharmacy Cluj-Napoca, 400337 Cluj-Napoca, Romania

**Keywords:** Atypical OPA, Epidemiology, Jaagsiekte sheep retrovirus type 2, Myxoid growths, Ovine pulmonary adenocarcinoma

## Abstract

**Background:**

Ovine pulmonary adenocarcinoma (OPA) is a neoplastic disease caused by exogenous Jaagsiekte Sheep Retrovirus (exJSRV). The prevalence of JSRV-related OPA in Eastern European countries, including Romania is unknown.

We aimed to investigate: the prevalence and morphological features of OPA (classical and atypical forms) in the Transylvania region (Romania), the immunophenotype of the pulmonary tumors and their relationships with exJSRV infection. A total of 2693 adult ewes slaughtered between 2017 and 2019 in two private slaughterhouses from Transylvania region (Romania) was evaluated. Lung tumors were subsequently assessed by cytology, histology, immunocytochemistry, immunohistochemistry, electron microscopy and DNA testing.

**Results:**

Out of 2693 examined sheep, 34 had OPA (1.26% prevalence). The diaphragmatic lobes were the most affected. Grossly, the classical OPA was identified in 88.24% of investigated cases and the atypical OPA in 11.76% that included solitary myxomatous nodules. Histopathology results confirmed the presence of OPA in all suspected cases, which were classified into acinar and papillary types. Myxoid growths (MGs) were diagnosed in 6 classical OPA cases and in 2 cases of atypical form. Lung adenocarcinoma was positive for MCK and TTF-1, and MGs showed immunoreaction for Vimentin, Desmin and SMA; Ki67 expression of classical OPA was higher than atypical OPA and MGs. JSRV-MA was identified by IHC (94.11%) in both epithelial and mesenchymal cells of OPA. Immunocytochemistry and electron microscopy also confirmed the JSRV within the neoplastic cells. ExJSRV was identified by PCR in 97.05% of analyzed samples. Phylogenetic analysis revealed the presence of the exJSRV type 2 (MT809678.1) in Romanian sheep affected by lung cancer and showed a high similarity with the UK strain (AF105220.1).

**Conclusions:**

In this study, we confirmed for the first time in Romania the presence of exJSRV in naturally occurring OPA in sheep. Additionally, we described the first report of atypical OPA in Romania, and to the best of our knowledge, in Eastern Europe. Finally, we showed that MGs have a myofibroblastic origin.

## Background

Ovine pulmonary adenocarcinoma (OPA) represents a virus-related neoplastic disease caused by an exogenous betaretrovirus [1]. The disease was first reported in South Africa in the late 1800s as an important cause of chronic respiratory distress in sheep [2]. The etiological agent of OPA is exogenous Jaagsiekte Sheep Retrovirus (exJSRV). A specific U3 long terminal repeat (LTR) sequence of exJSRV was detected in lungs from affected animals [3, 4]. The LTR region varies among different retroviruses, including JSRV [5], sheep endogenous retrovirus [3] and ovine enzootic nasal tumour virus [6]. Based on the U3 sequence and restriction profiles of the virus, some authors suggest that there are two types of exogenous retroviral sequences: type I (Kenyan and South African) and type II (Wyoming, USA and UK isolates) [3, 7]. ExJSRV has a specific tropism for the differentiated epithelial cells (type II pneumocytes and nonciliated bronchiolar Clara cells) of the lung, and it is the only virus known to cause pulmonary adenocarcinoma in naturally infected animals [8]. ExJSRV is mainly transmitted by infected aerosols [9, 10], and in natural cases the incubation period is very long, ranging from 2 to 4 years. Therefore it is most often encountered in adult sheep [11], but the lambs can also manifest clinical signs [6]. There is no evidence for breed or sex related OPA susceptibility [12]. Other animal species, including goats and moufflons were occasionally diagnosed with OPA [9, 13].

OPA shares many histological similarities with the human pulmonary adenocarcinoma, representing an important animal model for understanding the mechanisms of viral oncogenesis [14]. Although JSRV was found in human pulmonary neoplastic cells, its role in the development of lung cancer in humans is not fully elucidated [15].

There are two recognized forms of OPA which show gross, histological and immunohistochemical differences [4, 12, 16]. The lesions in classical OPA predominantly affect all pulmonary lobes and are located in the cranioventral area. They can be either nodular or exhibit a diffuse growth type, showing a grey moist appearance on the cross section. Atypical OPA consists of hard nodules, pearly-white that have a dry cut surface; the tumors are well-delimited by the surrounding pulmonary tissue [12].

The classical form is more common than the atypical type. In Europe, the classical OPA has been reported in several countries, such as: Ireland [17], UK [18], Scotland [19], Italy [20], Germany [21], Spain [22]. The incidence of the disease is usually low, but in some geographical areas it reaches up to 30% [23]. More than 50% of the affected animals usually die because of progressive respiratory failure [24], therefore causing important economic losses. The atypical OPA is less contagious than classical form [4]. The atypical OPA has been reported in Spain, Peru, Iran and India [4, 12, 16, 25], but there is no evidence of its occurrence in other countries where OPA is commonly found.

Clinically, the affected sheep develop chronic and progressive respiratory distress, especially when exercised. A common sign of classical OPA is mucous nasal discharge because of production of large fluids amounts in the lung [6]. This fluid might be absent in some cases, particularly in the atypical form of OPA, where the tumors remain incidentally found in abattoir studies or when the animals are necropsied for other reasons [12].

Currently, there are no efficient methods to clinical diagnose the disease and a full diagnosis can be obtained only post-mortem by histological examination [8]. Polymerase chain reaction (PCR) analysis of bronchoalveolar lavage (BAL) samples collected from living animals may be useful for pre-clinical identification of infected individuals with exJSRV. However, this technique is not able to identify all the early stages of classical OPA and atypical form, where the mucus production is absent or fewer infected cells are present in the BAL sample [21, 23]. Currently, this method is not extensively used because the sample collection requires sedation. Furthermore, *intravitam* diagnosis of exJSRV infection by PCR from blood offers many false negative results probably because the numbers of infected white blood cells is very low [26, 27]. Additionally, the viral genetic material or proteins can also be detected by PCR and immunohistochemistry (IHC) respectively, in pulmonary and lymphoid tissues [16, 17, 28].

Romania is an important European country in sheep farming, with over 12 million heads reported in 2018 according to Romanian Minister of Agriculture. There are only two studies regarding OPA presence in Romania with reported incidences of 0.5 and 0.8% [29, 30]. However, no evidence of exJSRV infection in relation with OPA, nor atypical OPA had been previously described. Currently, no information about the prevalence of classical OPA in Romania is available. Furthermore, to the author’s knowledge, exJSRV-related OPA has not been reported so far in other countries from Eastern Europe in the last decade.

In this study we aimed to investigate: 1) the prevalence and morphological features of OPA, classical and atypical forms, in Turcana sheep breed in the Transylvania region (Romania); 2) immunohistochemical features of the neoplastic epithelial components and myxoid growths (MGs), and 3) identification of exJSRV by electron microscopy, immunocytochemistry, IHC and PCR methods. A comparison between nucleotide sequences of exJSRV identified in sheep lung tumors and other reported exJSRV strains from different geographical regions was also previewed, in order to identify the strain present in Romania.

## Results

### Prevalence, distribution patterns and gross features of OPA

Out of 2693 examined slaughtered ewes, 45 cases were suspected of OPA after gross examination. However, histological examination confirmed the presence of OPA in 34 cases, therefore a prevalence of 1.26% of the disease in Transylvania (Romania). Data of sheep included in the study and confirmation of JSRV infection are summarized in Table 1.

**Table 1:** Data of sheep included in the study and confirmation of JSRV infection

Case samples	Breed	Gender/age*	Pathologic form	Histological type	IHC-JRSV	PCR-JSRV	ICC	TEM
1/15MAY17	Turcana	F	Classical	Papillary	+	+	NP	NP
2/25MAY17	Turcana	F	Classical	Papillary	+	+	NP	NP
3/56JUN17	Turcana	F	Atypical	MGs	+	+	NP	NP
4/33DEC17	Turcana	F	Classical	Acinar	–	+	NP	NP
5/34DEC17	Turcana	F	Classical	Acinar	+	+	NP	NP
6/36DEC17	Turcana	F	Classical	Acinar	+	+	NP	NP
7/02JAN18	Turcana	F	Atypical	Acinar	–	–	NP	NP
8/03JAN18	Turcana	F	Classical	Acinar	+	+	NP	NP
9/012JAN18	Turcana	F	Classical	Acinar	+	+	NP	NP
10/1JAN18	Turcana	F	Atypical	Acinar	+	+	NP	NP
11/2JAN18	Turcana	F	Classical	Papillary	+	+	NP	NP
12/5JAN18	Turcana	F	Classical	Papillary	+	+	NP	NP
13/10JAN18	Turcana	F	Classical	Acinar	+	+	NP	NP
14/3JUL18	Turcana	F	Classical	Papillary	+	+	NP	NP
15/5JUL18	Turcana	F	Classical	Acinar + MGs	+	+	NP	NP
16/6JUL18	Turcana	F	Classical	Papillary	+	+	NP	NP
17/7JUL18	Turcana	F	Classical	Acinar	+	+	NP	NP
18/02AUG18	Turcana	F	Classical	Papillary	+	+	NP	NP
19/03AUG18	Turcana	F	Classical	Papillary + MGs	+	+	NP	NP
20/1AUG18	Turcana	F	Classical	Acinar + MGs	+	+	NP	NP
21/2AUG18	Turcana	F	Classical	Papillary	+	+	NP	NP
22/3AUG18	Turcana	F	Classical	Acinar	+	+	NP	NP
23/5AUG18	Turcana	F	Classical	Acinar	+	+	NP	NP
24/6AUG18	Turcana	F	Atypical	MGs	+	+	NP	NP
25/1OCT18	Turcana	F	Classical	Acinar	+	+	+	NP
26/2OCT18	Turcana	F	Classical	Papillary + MGs	+	+	+	NP
27/3OCT18	Turcana	F	Classical	Acinar	+	+	+	–
28/4OCT18	Turcana	F	Classical	Acinar	+	+	+	–
29/5OCT18	Turcana	F	Classical	Acinar	+	+	+	NP
30/6OCT18	Turcana	F	Classical	Acinar + MGs	+	+	+	NP
31/7OCT18	Turcana	F	Classical	Acinar	+	+	+	–
32/8OCT18	Turcana	F	Classical	Acinar	+	+	+	NP
33/3MAY19	Turcana	F	Classical	Papillary + MGs	+	+	+	+
34/4MAY19	Turcana	F	Classical	Acinar	+	+	+	+

Age* - adult sheep (2–6 years); *F* female, *MGs* myxoid growths, *NP* not performed

In the remaining cases (*n* = 11), the diagnoses of chronic suppurative bacterial bronchopneumonia and verminous bronchopneumonia associated with extensive fibrosis and atelectasis were histologically differentiated from OPA. All diagnosed sheep belonged to Turcana breed and were adult females, the age ranging from 2 to 6 years.

**Fig. 1:**
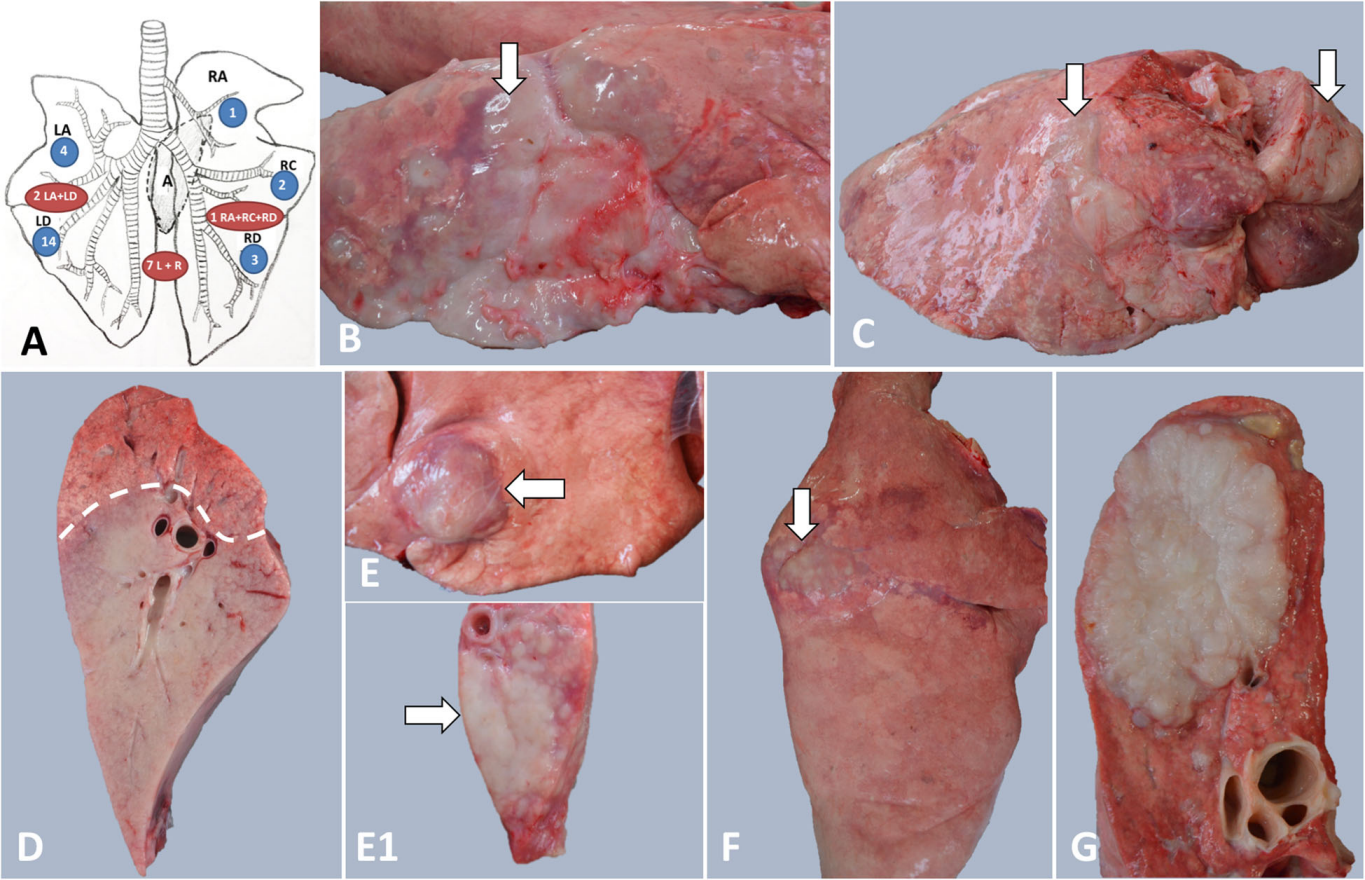
Gross features of spontaneous OPA in sheep. **a** Distribution of pulmonary lesions; **b** Classical form of OPA, involving the diaphragmatic lobe (arrow); **c** Classical form of OPA, affecting multiple pulmonary lobes (arrows); **d** Classical form of OPA on cut surface showing the junction (interrupted line) between the tumor and normal tissue; **e** and **e1** Atypical form of OPA showing a subpleural white-grey nodule (arrow). On cut surface, the tumor is pearly white and dried (arrow); **f** Gross aspects of pulmonary myxoma-like nodule (arrow); **g** the myxomatous mass shows a multilobular, white-gray, gelatinous feature on cut surface

On post-mortem examination, the distribution patterns of pulmonary lesions were divided into three major groups (Fig. 1a). Group I: in 20 cases (58.82%), the lesions were focal or solitary, multifocal to coalescing and diffuse (large masses), affecting a single pulmonary lobe with higher prevalence in the left diaphragmatic lobe (14 cases) (Fig. 1b). Group II: in 7 cases (20.59%), the lesions were located unilateral, but affecting 2 or more pulmonary lobes (Fig. 1c). Group III: in the other 7 cases (20.59%), neoplastic processes were bilaterally located, mainly affecting the diaphragmatic lobes and involving up to 80% of the pulmonary parenchyma (Fig. 1d). In these severe cases the lungs failed to collapse, and they were heavy and dense.

Grossly, the morphological features of pulmonary lesions were classified according to Garcia**-**Goti (2000) [4] into two main pathologic forms: classical OPA and atypical OPA. The classical OPA was identified in 30 cases (88.24%) and consisted of pink to light grey or white solitary nodules of varying size (1-5 cm) or large, dense confluent masses, reaching up to 30 cm in diameter. In some cases, the neoplastic masses were delimited from the normal parenchyma by a fine line of atelectasia or by a zone of emphysema. On the cut surface, the tumors included in this form were moist, had a homogenous appearance and the airways usually contained a viscous frothy fluid (Fig. 1d). In two cases, large areas of lytic necrosis and cavitation were randomly distributed within the neoplastic tissue. In most cases, multifocal to diffuse pleural fibrosis over the neoplastic masses was a common gross finding.

The atypical OPA was encountered in four cases (11.76%) and characterized by white, dried nodular or confluent lesions of approximately 2–2.5 cm in diameter, mainly located in the subpleural area of the left diaphragmatic lobe (Fig. 1e and e1). No or small amount of lung fluid was detected in these cases. In two of the cases, the pulmonary lesions consisted of white-grey, solitary, well delimited and unencapsulated nodules (Fig. 1f); on the cut surface, these nodules showed a multilobular, soft to dense and gelatinous appearance and consisted with myxoma-like masses (Fig. 1g).

### Cytological and histopathological features

**Fig. 2:**
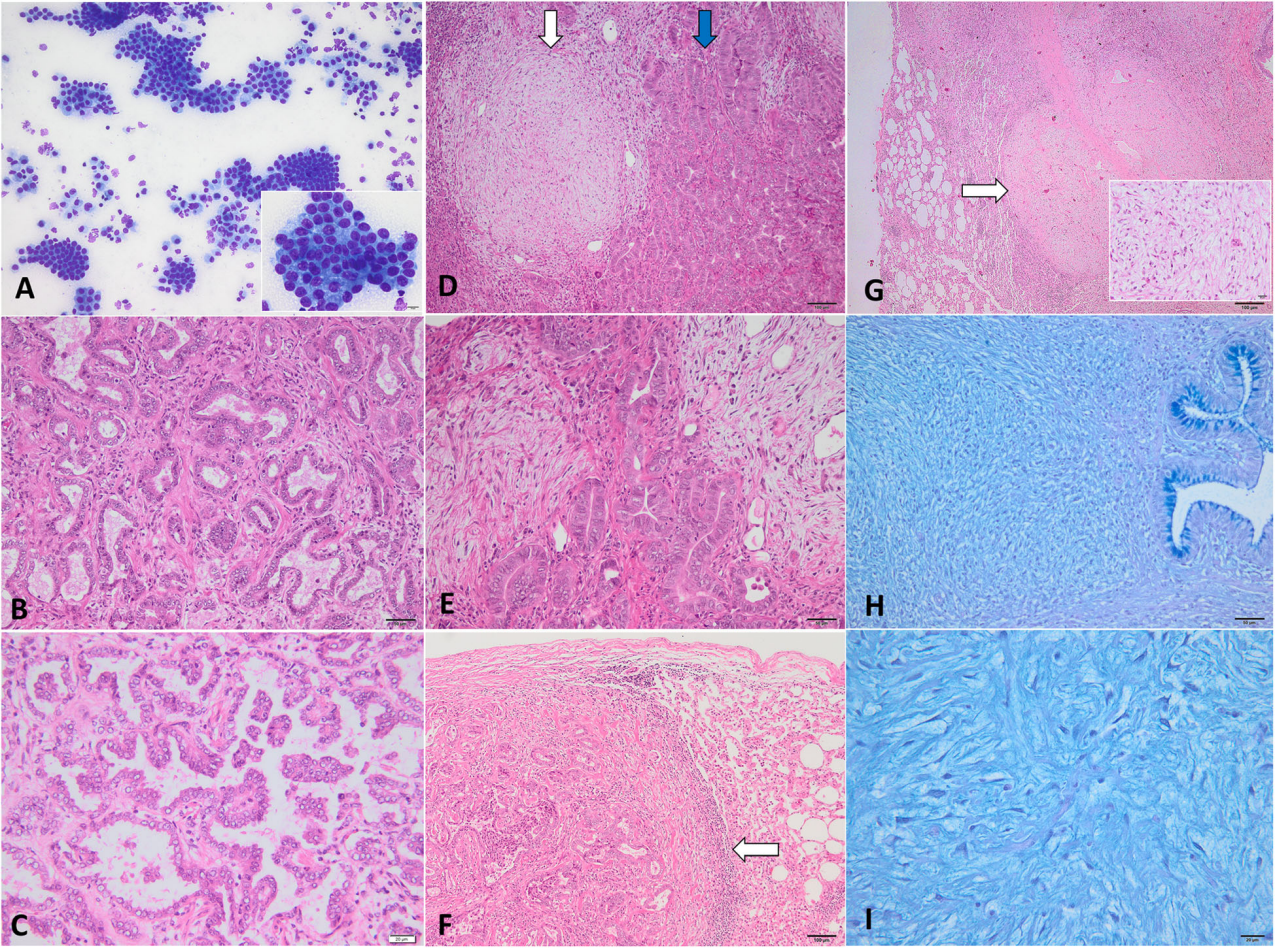
Microscopical findings of OPA. **a** Cytological exam of pulmonary tumors showing multiple nest of cubical to polygonal epithelial cells, interpreted as type II alveolar cells, DQ stain; **b** Histological appearance of OPA-classical form: tubular type and **c** papillary type, H&E stain; **d** and **e** The microphotographs of classical OPA showing myxoid growths (white arrow) interspersed with neoplastic epithelial component (blue arrow), H&E stain; **f** Histological exam of atypical OPA showing a well-delimited neoplastic nodule, surrounded by fibrous tissue and infilammatory infiltrates (arrow), H&E stain; **g** Myxoma-like tumor without neoplastic epithelial component. The mass is multinodular (white arrow) and composed of spindle to stellates cells, and abundant extracellular matrix (the inset), H&E stain; **h** and **i** AB-PAS stained sections from myxoma-like nodules showing abundant, pale blue extracellular myxomatous matrix; the Goblet cells of bronchial lining epithelium are used as positive control for AB-PAS stain

Cytological examination of both classical and atypical forms of OPA revealed numerous well differentiated cuboidal or polygonal neoplastic epithelial cells arranged in small acini, clusters or individually (Fig. 2a). The neoplastic cells had a moderate amount of pale blue, finely granular cytoplasm, moderate nuclear: cytoplasmic (N/C) ratio and round to oval, centrally located, nuclei with finely stippled chromatin and 1–2 distinct blue nucleoli. Anisocytosis and anisokaryosis were mild to moderate with rare mitotic figures. Large numbers of macrophages and mature lymphocytes were admixed with the neoplastic cells. OPA containing MGs were poorly cellular and composed of spindle, stellate and elongated cells with round to oval nuclei, and inserted in a pale blue slightly vacuolar extracellular myxoid material. All tumors diagnosed by cytological exam were subsequently confirmed by histopathology and immunohistochemistry.

Histologically, all pulmonary tumors classified as classical form of OPA (*n* = 30), were composed of a single layer of neoplastic cuboidal, columnar or polyhedral epithelial cells lining the alveolar lumina or bronchioles, surrounded by a fine to moderate fibrovascular stroma. The predominated histological types of pulmonary adenocarcinoma were represented by acinar (*n* = 22) (Fig. 2b) and papillary (*n* = 8) (Fig. 2c). The diameter of individual neoplastic epithelial cells ranged from 20 to 30 μm, showed moderate amount of pale acidophilic, finely granular cytoplasm and intermediate N/C ratio. Anisokaryosis and anisocytosis were low to moderate; the nuclei were round to oval, centrally located, with a fine granular to lacy chromatin with and 1–2 distinct, basophilic nucleoli. The average of mitotic rate was 2 per 10 HPF without atypical features. Multifocal, the stroma was heavily infiltrated with macrophages, lymphocytes and few plasma cells. The adjacent parenchyma of the neoplastic mass showed atelectasis, and at the periphery of the tumors, the neoplastic cells were occasionally arranged in a lepidic growth pattern. The myxoid growths consisting of sparsely cellular structures of spindles cells embedded in an abundant extracellular matrix were observed in 6 cases of classical OPA. The MGs were admixed with the neoplastic epithelial component, or in some areas were disposed as multiple poorly demarcated masses (Fig. 2d, e).

The histological features of the atypical form (*n* = 4) were similar to those described in classical form, and two cases were classified as acinar type. The distinguish of atypical OPA features were represented by more prominent limits between normal tissue and neoplastic masses due to higher fibroblast proliferation and inflammatory infiltrates, predominated by mature lymphocytes and macrophages (Fig. 2f).

In the other two cases of pulmonary solitary nodules, the neoplastic epithelial component was absent, and the neoplasm was composed by short bundles and streams of spindle, stellate or individual cells (Fig. 2g). The mesenchymal cells were embedded into an abundant myxoid matrix that contains moderate amounts of foamy, amphophilic, AB-PAS positive material (mucin) (Fig. 2h and i). The individual cells had variable distinct borders, intermediate N/C ratio and moderate amounts of pale acidophilic, homogenous cytoplasm. The nuclei were oval to elongated, 20–30 μm in diameter, in transverse section, centrally located with clumped chromatin and indistinct nucleoli. No mitotic figures were present; anisocytosis and anisokaryosis were low. Based on the histological features, a presumptive diagnosis of pulmonary myxomas was made.

Overall, mesenchymal proliferations and myxomatous changes, named as myxoid growths (MGs), were identified in 8 out of 34 (23.52%) tumors: 6 cases of classical form and 2 cases of atypical form consistent with pulmonary nodules without epithelial cell neoplasia (myxoma-like nodules).

### Immunohistochemical features of pulmonary masses

**Fig. 3:**
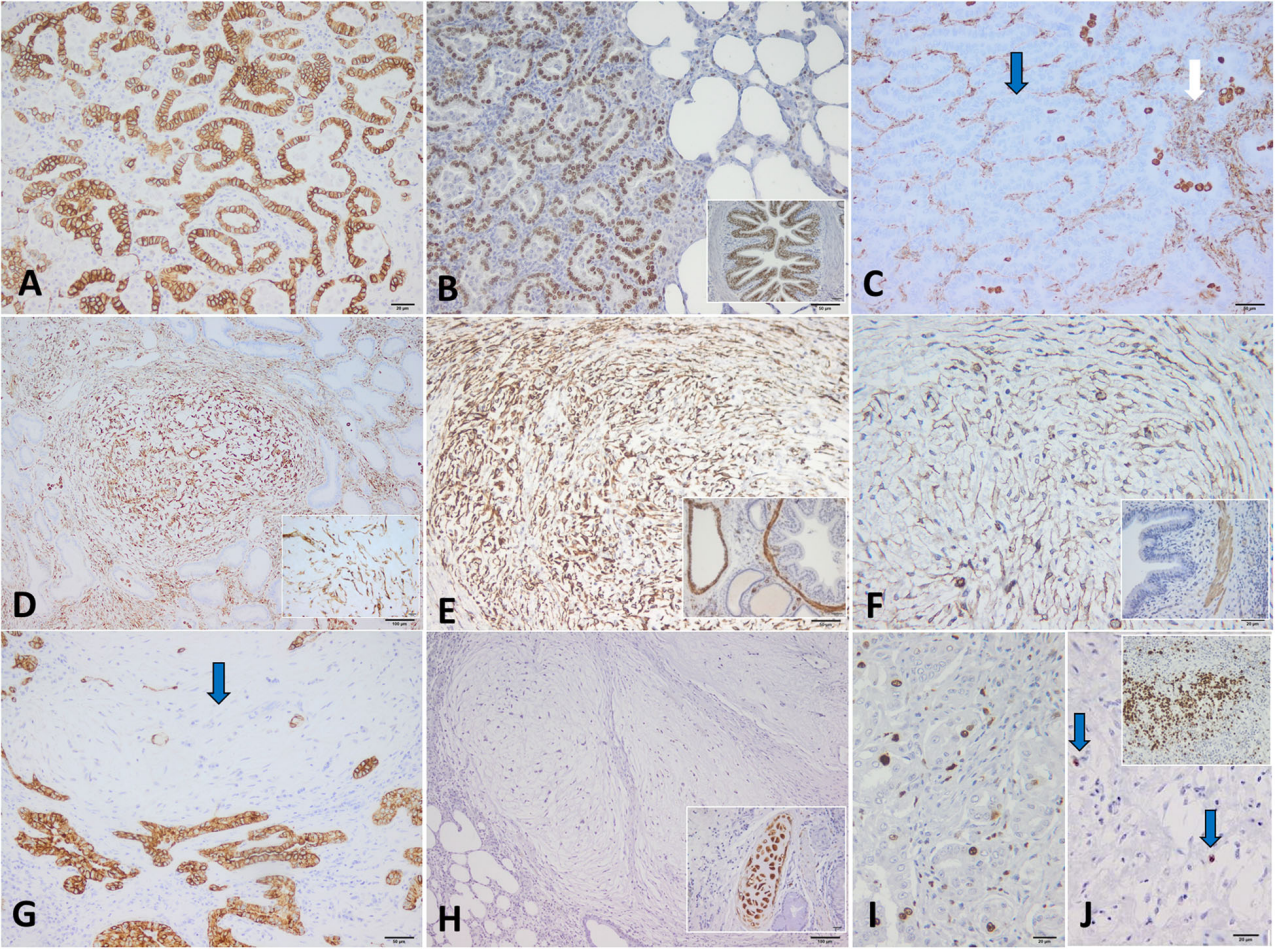
Immunophenotypical characterization of OPA. **a** The neoplastic epithelial cells of classical form are diffusely and intensely immunopositive for MCK; **b** TTF1-positive nuclei of the epithelial cells are present within the neoplastic masses. Inset: the bronchial epithelium was used as positive control for TTF1. **c** All epithelial cells lining neoplastic acini are negative for vimentin (blue arrow) in contrast to immunopositive stromal cells (white arrow); **d** Myxoid growths showing an intense immunopositive reaction for Vimentin. Inset: detail of diffuse cytoplasmic labeling for Vimentin; **e** Mesenchymal cells of myxoid growths have diffuse and strong cytoplasmic labeling for Desmin. Inset: The smooth muscle cells of pulmonary arteries and bronchioles are strongly positive for desmin and served as internal positive control; **f** The cells of myxoid growths have selective and moderate cytoplasmic labeling for alpha-SMA. Inset: The bronchiolar smooth muscle cells are positive for SMA, internal positive control; **g** The myxoid component of OPA showing a negative immunoreaction of MCK (blue arrow) compared to the neoplastic epithelial cells which are strongly immunopositive; **h** Neoplastic cells of myxoma-like nodules showing a negative reaction for S100 protein. Inset: the bronchial cartilage is diffusely positive for S100 protein, internal positive control; **i** The proliferative index, characterized by ki67 immunopositive nuclei, was higher in the epithelial neoplasia compared to the MGs and myxoma-like nodules (blue arrows) (**j**). Inset: Bronchial-associated lymphoid tissue (BALT) hyperplasia was used as internal positive control for ki67. DAB and hematoxylin counterstain

Immunohistochemically, in both forms of OPA, the neoplastic epithelial component showed a strong and diffuse reaction for MCK (Fig. 3a) and TTF-1 (Fig. 3b); the epithelial cells were negative for vimentin (Fig. 3c). The cells of MGs were diffusely and intense positive for vimentin (Fig. 3d), desmin (Fig. 3e), selective positive for alpha-SMA (Fig. 3f) and negative for MCK (Fig. 3g), suggesting their true mesenchymal origin, probably from pulmonary interstitial myofibroblasts. All mesenchymal components were also negative for S100 protein (Fig. 3h) and TTF1. The proliferative index (ki67) of the epithelial component of classical OPA was higher (mean value of 10.87%) (Fig. 3i) than atypical OPA (mean value of 4.54%), whereas in the MGs, this labelling was low in all forms (mean value of 1.61% classical form and 2.48% in atypical OPA, consisting of myxoma-like nodules without neoplastic epithelial component) (Fig. 3j).

### JSRV identification methods

#### Immunocytochemistry and immunohistochemistry

**Fig. 4:**
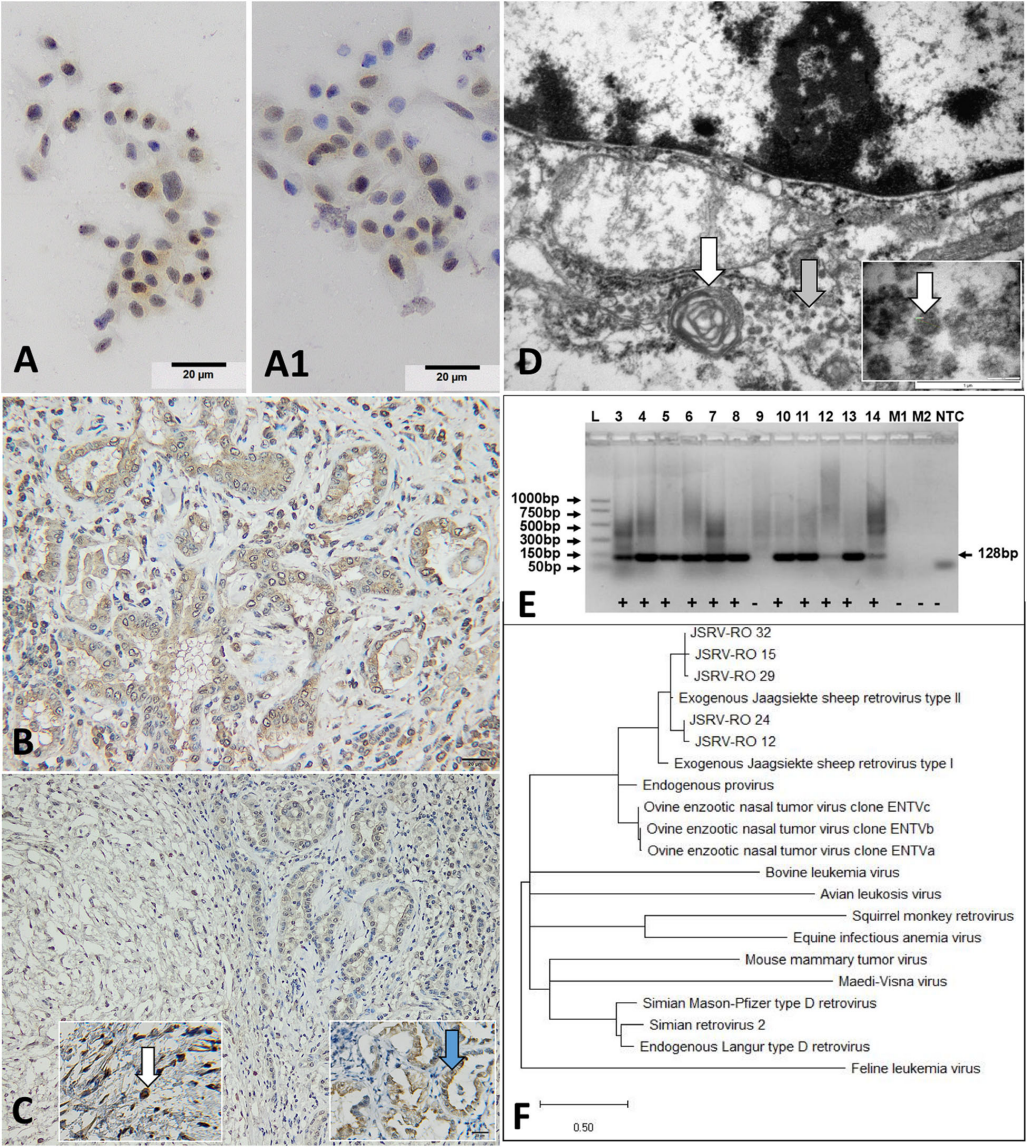
JSRV detection from neoplastic tissues. **a** and **a1** Immunocytochemistry showing intracytoplasmic JSRV-MA expression within the neoplastic epithelial cells. DAB and hematoxylin counterstain; B Diffuse expression of the JSRV-MA marker by neoplastic epithelial cells forming tubular structures; **c** Expression of the JSRV-MA marker by MGs and epithelial tumor. Left inset: Cell cytoplasm of MGs (white arrow) is strongly labelled with JSRV-MA antibody. Right inset: Neoplastic epithelial cells have diffuse cytoplasmic labelling for JSRV-MA (bue arrow). DAB and hematoxylin counterstain; **d** JSRV-infected type 2 pneumocytes. Note the presence of intracytoplasmic lamellar body (white arrow) and aggregates and solitary intracytoplasmic viral particles (grey arrow). TEM. Bar, 1 μm. Inset: Detail of a viral replication site showing specific virions compatible with JSRV (white arrow). TEM. Bar, 100 nm; **e** Electrophoresis profile of the amplified DNA fragments (128 bp) evidencing the presence of proviral Jaagsiekte DNA in ovine pulmonary adenocarcinoma (OPA). L - DNA ladder; PCR positive (+) samples; PCR negative (−) samples; M1 and M2 control samples amplified from healthy ovine lung tissue; NTC - no template control (negative control); **f** Phylogenetic analysis of different JSRV strains and other retroviruses based on the LTR region. The Romanian strains of exJSRV (JSRV-RO) (GenBank MT809678.1) showed homology at nucleotide level of 97% with UK strain (GenBank AF105220.1), and 83% with the South African strain (GenBank M80216)

Immunocytochemical staining of imprints smears showed a positive reaction consistent with the presence of intracytoplasmic JSRV antigen within the neoplastic cells (Fig. 4a and a1). No immunocytochemical expression of JSRV-MA was detected in the imprints smears from normal lungs.

In both classical and atypical OPA, immunohistochemical evaluation of pulmonary tumors showed a diffuse positive JSRV-MA reaction of neoplastic cells in 32 cases (94,11%). The positive reaction was characterized by a finely granular brown staining of the cellular cytoplasm of both neoplastic epithelial component (Fig. 4b) and MGs (Fig. 4c). Additionally, a positive reaction was also observed for the inflammatory cells, mainly macrophages and lymphocytes. Moreover, the intensity and number of JSRV-positive cells in atypical tumours were reduced compared to the classical form of OPA. A negative JSRV-MA reaction was found in all normal pulmonary tissues (*n* = 10), selected as negative control.

#### Transmission electron microscopy (TEM)

Ultrastucturally, the neoplastic type II pneumocytes have been recognized by the presence of microvilli, basal or centrally located nuclei, numerous intracytoplasmic, moderately electronodense, round and lamellar structures of approximately 350–400 nm in diameter, interpreted as lamellar bodies (Fig. 4d). Additionally, round oval structures of 1–2 μm in diameter, delimited by a moderately electron-dense membrane and containing an abundant electron lucent material (intracytoplasmic vacuoles) were also identified within the cytoplasm of type II alveolar cells. JSRV particles were found within the aforementioned intracytoplasmic vacuoles or in the cytosol of the neoplastic cells, in 2 out of 5 examined samples. The viral particles were arranged into small groups (4–6 virions) or individually, measured about 80–120 nm in diameter, and showed a moderately electron-dense central area (Fig. 4d). Extracellular JSRV particles were not observed in none of the examined examples.

#### PCR, DNA sequencing and phylogeny

All pulmonary lesions, histologically diagnosed as OPA, were submitted for PCR analysis. The expected 128 pb amplicons (obtained from exJSRV proviral DNA) were visualized in 33 out of 34 analysed samples (97.05%), while no bands were visible in the negative controls (Fig. 4e). The unaffected tissues adjacent to the neoplastic masses were also negative for the virus presence. Based on the obtained LTR nucleotide sequences of JSRV (deposited in GenBank under accession number MT809678.1) from lung tumours and their comparison with other retroviral strains available in GenBank, we evidenced a homology at nucleotide level of 97% with exJSRV UK strain (GenBank, AF105220.1), and only 83% with the South African strain (GenBank, M80216) (Fig. 4f). Therefore, we concluded that affected animals are infected with Jaagsiekte Sheep Retrovirus type 2, which is the most probable responsible for the adenocarcinoma found in Romanian Turcana sheep.

## Discussions

Primary respiratory tract and lung tumors in animals have a lower incidence compared with other systems, as well as compared to human patients. In contrast, the lung is the preferential site of metastases in all animals [31]. In sheep, the most common tumor of the respiratory system is ovine pulmonary adenocarcinoma (OPA), also known as jaagsiekte or ovine pulmonary adenomatosis [32].

This work confirms the presence of JSRV-infected sheep in Romania. To the author’s knowledge, JSRV-related OPA has not been reported in other countries of Eastern Europe in the last decade.

For this study, the samples were collected from two slaughterhouses which are representative for Transylvania, not for the whole country. Unfortunately, due to specific slaughtering procedures, we collected the pulmonary tissue samples only at the end of slaughtering. As a consequence, an exact match between examined lungs and ear tag for each slaughtered sheep was not possible to be determined. Therefore, neither the exact origin and age of each sheep were not possible to be established. However, according to the records of the slaughterhouses, all slaughtered individuals were adult females and originated from different counties of Transylvania, including Cluj, Bistrita-Nasaud, Mures, Sibiu and Alba. Thus, the authors can relate that all the positive OPA cases originated from Transylvania, a historical region that is located in center of Romania. The present study shows that the prevalence of OPA is about 1.26%, and is higher compared to the previous reports regarding OPA situation in Romania, 0.8 and 0.5%, respectively [29, 30]. ExJSRV presence was not determined in any of these two previous studies. Furthermore, the prevalence of OPA in Romania is higher than in other European countries, including UK (0.9%) [18] and Ireland (0.5%) [17]. We can think about two possible explanations of this prevalence of OPA in Romanian Turcana sheep. First, it can be related with a high number of sheep that are raised in Romania. According to the Ministry of Agriculture data the number of sheep in 2019 was over 11 million heads. This places the Romanian sheep industry in the third position in Europe after Spain and UK. Another important causative factor could be related with sheep management in Romania. The majority of animals are raised in free pastures and there is a lot of sheep movements between regions to find fresh grasslands. This is a traditional sheep breeding management and it is a way of life for the sheep breeders that is still very present in Romania. These two factors might explain a higher OPA prevalence in Romanian sheep compared with other European countries. In contrast to the prevalence of OPA in Romania, the disease is quite common in South America, South Africa, and Scotland, where 5–20% of infected animals develop pulmonary tumors [33].

OPA represents a continuous issue regarding proper methods of diagnosis and prevention [34, 35]. Despite some specific clinical signs of classical OPA, (including dyspnea and tachypnea), in combination with progressive weight loss and nasal mucous discharge [36], and suggested imaging modalities (radiography, computed tomography and ultrasonographic examination) [37, 38] currently the gold standard diagnostic method for both classical and atypical OPA relies in gross and histology exams performed during post mortem evaluation [7]. In our study, data about clinical signs of slaughtered sheep with OPA were not available. Postmortem evaluation, including gross and histological exams of the affected sheep is essential for confirmation of OPA. Although gross changes are relatively specific for OPA, in some cases chronic bronchopneumonia should be included in the differential list. A recent study showed that numerous cases (78%) of suspected OPA, based on gross changes, were false positive lesions due to its similarities with chronic bronchopneumonia; moreover 67% were exJSRV-negative by RT-PCR and IHC. In some cases, OPA and bronchopneumonia can occur concurrently, and the pneumonic lesions may obscure the characteristic histological features of OPA [17]. Lesions of chronic bronchopneumonia in sheep are usually locally-extensive, dense, grey-dark red in colour, affecting the cranioventral region of the lung [33]. In our study, chronic suppurative bacterial bronchopneumonia without neoplastic lesions was histologically diagnosed only in four cases of suspected OPA, characterized by cranioventral consolidation of the pulmonary lobes. Our findings showed that the distribution of both classical and atypical forms of OPA was different than those caused by chronic bacterial bronchopneumonia, and the diaphragmatic lobe was the most affected part of the pulmonary parenchyma. In the other two cases, OPA had concurrent pulmonary necrosis, fibrosis, atelectasis and abscesses caused by *Corynebacterium pseudotuberculosis*, and these findings are in agreement with a previous study [18].

The histological features of OPA consist of proliferation of epithelial cells into acini, papillary and solid structures, supported by a fibrovascular stroma. In atypical form, in addition to the epithelial neoplasia, marked fibroblast proliferation, collagen deposition and mononuclear cell infiltration are characteristic microscopic changes [39]. These inflammatory changes may be caused by intercurrent infections or a specific immune response of the host [4]. Occasionally, small nodules of neoplastic loose mesenchymal tissue appear admixed with the neoplastic component [40]. Both epithelial and mesenchymal components showed rare mitoses and a ki67 index mean value of 11.4% [41], and this is in good agreement with our findings, where ki67 index of the epithelial component of classical OPA was higher (mean value of 10.87%) than atypical OPA (mean value of 4.54%).

Another subtype of solitary tumor associated with exJSRV in sheep is grossly characterized by round or elongated, white–grey nodules of bright gelatinous appearance, and sharply demarcated from the surrounding parenchyma [41]. These nodules are histologically classified as myxoid nodules or myxoid growths or mesenchymal growths (MGs) [12, 41]. MGs resemble characteristic features of benign mesenchymal tumors and have been described in a variable proportion of tumours, intermingled with the neoplastic epithelial component [12]; in some cases, they were identified in metastases [22]. The MGs are not an uncommon feature encountered in OPA, but interestingly, they were not reported in experimental studies [42].

In the current study, 8 out of 34 ovine pulmonary tumors presented MGs, accounting 23.53% of the samples, although there are reports of up to 40% of the OPAs that contain also the neoplastic mesenchymal component [43].

The MGs are also recognised of being associated to JSRV infection due to the positive constant virus immunolabeling [40, 41, 44]. Our results revealed, in all cases of MGs, an intense immunoexpression using antibodies against JSRV-MA, and confirmed that the MGs represent JSRV-associated neoplastic growths.

The MGs were immunohistochemically characterized using mesenchymal and muscle cells markers, but their embryonic origin is still debated. IHC showed that these nodules are composed of true mesenchymal cells, and do not express epithelial cell markers [41]. The histological features of MGs are similar to myxomas that have a population of spindle or stellate cells embedded in a matrix rich in acid mucopolysaccharides [45]. Primary pulmonary myxoma not associated to JSRV was also reported in sheep [46, 47]. In our study, two cases of pulmonary nodules showed specific gross, histological and histochemical (AB-PAS matrix) features of pulmonary myxoma, and were confirmed through IHC. Pulmonary myxomas are immunopositive for vimentin and positive for S100 protein [47]. Our results, regarding the negative immunoreaction of pulmonary myxomas and MGs for S100 protein are in contrast with these previous evidence [47], but go towards other reported data [46].

A recent study demonstrated that the origin of mixed solitary OPA, containing both epithelial and mesenchymal components, consisted of local progenitor cells involved in lung repair [41]. It is well known that a small population of contractile fibroblasts are frequently found in normal alveolar septae, and the myofibroblast overactivity is directly correlated with lung fibrosis progression [48]. In lungs chronically affected with fibrosis, the mesenchymal cell phenotypes vary from proliferating fibroblasts to fully differentiated smooth muscle cells, and the predominant cell type is the myofibroblast, a fibroblast-like cell that expresses alpha-smooth muscle actin (alpha-SMA) [49]. Moreover, an immunohistochemical study of human pulmonary fibrosis revealed that myofibroblasts express SMA, vimentin, and desmin [49, 50].

Based on the immunohistochemical results of the present study and according to the aforementioned findings, our hypothesis suggests that the cell origin of this mesenchymal proliferative lesions or MGs is most likely contractile myofibroblasts.

IHC is also a useful method to identify small pulmonary lesions of OPA and to differentiate primary form metastatic pulmonary tumors, but is not routinely used as commercial test in veterinary practice [17]. IHC was recently used for evaluation of classical and atypical form of OPA in sheep [16, 51].

In the present study all tumors diagnosed as acinar and/or papillary predominate type of pulmonary adenocarcinoma, were MCK and TTF1 positive, and negative for vimentin, suggesting a primary epithelial origin of lung tissue. TTF1 (Thyroid transcription factor-1) is a 38 kDa nuclear protein member of the Nkx2 homeodomain transcription factor family, expressed by canine and feline type II alveolar pneumocytes, bronchiolar and thyroid epithelial cells [52, 53]. In this population, TTF-1 expression was intensely and diffusely positive in all epithelial cells of pulmonary adenocarcinoma, classical and atypical forms, and negative in the MGs, confirming that TTF-1 maintains a very high specificity for respiratory epithelium in sheep, as suggested by previous studies [54].

The results of this study also provide important evidence on the use of ICC and TEM on JSRV identification in postmortem collected lung tissue. The cellular composition of spontaneously-arising OPA tumours has been previously characterized by electron microscopy as round oval virions of 100–125 nm in diameter, with small bump-like structures existing on the surface of the virus particles [55], and are confirmed by our findings. ICC is a highly productive method used in biomedical research to identify various antigens in cells [56]. In this study, ICC showed the presence of intracytoplasmic JSRV antigen within neoplastic cells and fewer macrophages in all examined cases. Furthermore, all ICC positive cases were confirmed by IHC and PCR analyses. This method could have many practical applications but, to the author’s knowledge no other data supporting the utility of ICC in the detection of JSRV in sheep is reported. We consider that ICC could be used as a rapid and efficient test for JSRV identification from OPA, and it could have a possible application for intravitam diagnosis of JSRV, however this requires further studies.

PCR method is considered to be more sensitive than IHC for identification of exJSRV from lung tissue samples [17]. In this respect, we aimed to detect by PCR the presence of exJSRV proviral DNA by amplifying a region from the LTR sequence. ExJSRV was identified by PCR in 97.05% of analysed samples exhibiting tumors, showing a higher sensitivity than immunohistochemistry (94.11%). In all cases, the adjacent pulmonary parenchyma was negative for exJSRV. However, in one case, we could not prove the presence of JSRV in OPA neither by PCR nor IHC. The PCR results are in agreement with those described in other studies [17].

Bai et al.*,* (1996) [3] demonstrated that exJSRVs derived from some endogenous JSRV loci following mutations in LTR or other regions of the proviral genome during evolution. The long terminal repeat (LTR) U3 sequence and the envelope gene (env) are the major factors of retroviral tropism. LTR contains the viral promoter and enhancer which interact with the cellular transcription machinery, and it is preferentially active in cell lines originated from Clara cells and alveolar type II pneumocytes [57, 58]. LTR is also considered the only element capable to distinguish exogenous JSRV associated with OPA from endogenous loci in the sheep genome [3]. In our report, the phylogenetic analysis showed that the Romanian isolates (JSRV-RO) belonged to exJSRV, type 2, and it was very similar to UK strain (accession number AF105220.1). These findings provide novel information on the geographic distribution of the genetic lineages of exJSRV in sheep.

Our findings confirm that exJSRV - related OPA represents a retroviral neoplastic disease that needs to be included in the list of possible differential diagnoses of chronic respiratory distress in the sheep population in Eastern Europe. The zoonotic potential of sheep exJSRV- OPA infection and its implications in human health remains to be investigated.

## Conclusions

The present study contributes to a better understanding of the epidemiology, cellular origin and morphological features of OPA, providing new insights into the geographical distribution of this infectious disease. In this study, we confirmed for the first time in Romania the presence of exogenous JSRV in naturally occurring OPA in a group of slaughtered Turcana sheep by imunocytochemsitry, immunohistochemistry, electron microscopy and PCR. Additionally, we described the first report of atypical form of OPA in Romania, and to the best of our knowledge, in Eastern Europe. We also demonstrated that the MGs are myofibroblastic in origin. Our data highlights the need for further research to establish the prevalence of OPA all over the country and to develop national monitoring programs.

## Methods

### Tissue samples

Out of 2693 examined adult ewes slaughtered between 2017 and 2019 in two private slaughterhouses located in Bistriţa-Năsăud and Sibiu counties from Transylvania region of Romania, thirty-four individuals were identified with macroscopic lesions resembling lung tumors. All neoplastic lesions were subsequently collected for laboratory investigations. Normal pulmonary tissues were also collected from ten animals free of disease. Both normal and neoplastic pulmonary samples were subdivided and either fixed in 10% buffered formalin for microscopy studies, or submerged in 2.7% glutaraldehyde solution for electron microscopy examination. Part of these tissues were stored at − 20 °C for subsequent molecular genetics studies. The inclusion criteria were breed (Turcana), origin of sheep (Transylvania region, Romania), age (adult ewes), and presence of pulmonary masses compatible with classical and atypical forms of OPA.

### Cytology and histopathology

Cytological examination was performed from twelve cases (ten of classical form and two of atypical form) by impression smears method and stained using Dia Quick Panoptic technique (DQP, Reagens Kft, Budapest, Hungary).

For histopathology, collected tissue samples were routinely processed for paraffin embedding by using graded concentrations of alcohol for dehydration and xylene. The paraffin blocks were cut at 2–3 μm thickness and stained with hematoxylin-eosin (H&E). In addition, for pulmonary myxoid growths, alcian blue-periodic acid Shiff stain (AB-PAS) was also performed. The tumors were classified according to World Health Organization for domestic animals’ diagnostic criteria [32, 59]. Pulmonary myxoid growths were described according to [41]. For both histology and immunohistochemistry, the sections were independently examined by two pathologists (MT and CT) using a light Olympus BX-41 microscope and the photomicrographs were taken using an Olympus SP 350 digital camera and Stream Basic imaging software (Olympus Corporation, Tokyo, Japan). When there was a divergence of opinion, an agreed diagnosis was reached through simultaneous evaluation in a multi-head microscope (Zeiss Axio Scope A1).

### Immunohistochemical evaluation of pulmonary masses

For the immunohistochemical study, a panel of primary antibodies specific for several antigens, including multi-cytokeratin (MCK), vimentin (Vim), alpha smooth-muscle actin (alpha-SMA), desmin, S100 protein, Ki67 and Thyroid Transcription Factor 1 (TTF-1) was applied (Table 2). The samples were automatically processed using Leica BondmaxTM Immunohistochemistry system (Leica Biosystems Melbourne, Bond Max model, M2 12,154 series). The positive reaction was given by the brown stain of the cytoplasm for MCK and desmin, cytoplasm and cellular membrane for Vim and SMA, cytoplasm and nuclei for S100, and nuclei for TTF-1 and Ki67. Positive controls were applied for each antibody. Negative controls were performed by replacing the primary antibody with normal serum from the same species as primary antibody.

**Table 2:** Antibodies used for ovine pulmonary tissues immunohistochemistry

Specificity of antibody	Clone	Provider	Dilution	Positive control
JSRV-MA	–	Prof. Massimo Palmarini	1/1500	OPA classical Tumour, PCR+
Multi-cytokeratin	AE1/AE3	LeicaBiosystems Newcastle Ltd	Ready to use	Bronchial epithelium
Vimentin	clone SRL33	LeicaBiosystems Newcastle Ltd	Ready to use	Endothelial cells
TTF-1	8G7G3/1	Cell Marque Milipore Sigma	1/200	Type II pneumocytes, bronchioles
Desmin	DE-R-11	LeicaBiosystems Newcastle Ltd	Ready to use	Smooth muscle
SMA	1A4	Abcam	1/200	Smooth muscle
S100	Polyclonal	LeicaBiosystems Newcastle Ltd	Ready to use	Bronchial wall cartillage
Ki-67	MM1	LeicaBiosystems Newcastle Ltd	Ready to use	Lymphoid tissue (BALT)

### JSRV identification methods

#### Immunocytochemistry and immunohistochemistry

Immunocytochemistry (ICC) was performed on all OPA suspected cases using a primary antibody against JSRV-MA. The samples were obtained using the touch imprint method from the neoplastic masses and the adapted protocol for IHC using Novolink™ Polymer Detection System kit (Leica Biosystems). Briefly, cytological smears were fixed in 95% ethanol, followed by protein block, peroxidase block, incubation with primary antibodies (1 h at room temperature) followed by visualization of the reaction using the avidin-biotin complex and 3, 3′-diaminobenzidine (DAB) chromogen (Novolink™ DAB, Leica Biosystems). The samples were counterstained with Mayer’s hematoxylin.

JSRV-MA was detected immunohistochemically using the standard manual protocol, according to the provider (Massimo Palmarini, Centre for Virus Research, University of Glasgow, Scotland). The paraffin sections were dewaxed in xylene, followed by rehydration in decreasing concentrations of alcohols. Epitope retrieval was performed in sodium citrate buffer (pH -6) followed by protein blocking using normal horse serum for 1 h. Endogenous peroxidase was blocked with peroxidase block for 5 min. The primary antibody was maintained overnight at 4 °C in a humid chamber, followed by placing the secondary antibody. Every step, except the one between protein blocking and primary antibody placement was followed by two washing steps in phosphate buffered saline. The reaction was visualised using DAB. The sections were stained with Mayer’s hematoxylin. The positive reaction was given by the brown labelling of the neoplastic cells.

#### Transmission electron microscopy

For transmission electron microscopy (TEM), 4 fragments of approximately 1 mm^2^ from both pulmonary masses and adjacent normal tissues were collected and maintained in 2.7% glutaraldehyde for 2 h. From every sample, two representative blocks were dissected. The samples were subsequently fixed in 2% osmium tetraoxide for 90 min at 4 °C, followed by dehydration in increasing concentrations of acetone and embedded in Epon 812 for 12 h. From every sample, two representative blocks were dissected. The encapsulation and polymerization of the samples were performed for 48 h at 60 °C; the samples were modeled and cut with a Leica UC7 ultramicrotome at 60–90 nm thickness and floated onto 200-mesh grids. Contrasting was performed using uranyl acetate and lead citrate stain, and the sections were examined using the Jeol 1010 electronic transmission microscope.

#### PCR, DNA sequencing and phylogeny

Genomic DNA was extracted from all collected fresh-frozen tissues samples (pulmonary masses and normal lungs) with the Quick-gDNA MiniPrep kit and according to the manufacturer instructions (Zymo Research Corporation, USA). PCR amplification for the proviral DNA identification was performed in 25 μl reactions containing: 12.5 μl GoTaq G2 PCR Master Mix (Promega, USA), 9.5 μl of nuclease-free water, 1 μl (10 pmol/μl) of each specific primer designed from the viral long terminal repeat (LTR) region and reported before [5], designated as exJSRV-F: 5′-TGGGAGCTCTTTGGCAAAAGCC-3′ and exJSRV-R: 5′- TGATATTTCTG TGAAGCAGTGCC - 3′. For each reaction 50 ng of genomic DNA was used, except for no template control reactions, in which ultrapure water was added instead of DNA. The thermal profile for PCR amplification consisted of 1 cycle at 95 °C for 3 min followed by 35 cycles at 94 °C for 30 s, 58 °C for 30 s, 72 °C for 30 s and a final extension step at 72 °C for 7 min. Part of the PCR reactions were checked for specific amplicons on 3% agarose gels stained with 1X SybrSafe (Invitrogen, USA) and analyzed under UV light.

Five PCR reactions exhibiting specific amplicons associated with the presence of exJSRV from two cases of classical OPA (JSRV-RO 15, JSRV-RO 29), 2 cases of atypical OPA (JSRV-RO 12, JSRV-RO 24) and one case of pulmonary myxoma-like nodule without epithelial component (JSRV-RO 32) were selected for sequencing. PCR primers and unincorporated nucleotides were removed with the ExoSap-IT cleanup reagent (Thermo Scientific, USA). Amplicons were bi-directionally sequenced with each PCR primer in separate reaction tubes, by employing the BigDye Terminator v3.1 Cycle Sequencing Kit (Thermo Scientific, USA). Sequencing products were further purified with the BigDye Xterminator Purification Kit and subsequently analyzed on an ABI 3500 device (Thermo Scientific, USA).

The obtained LTR sequences, and additional ones from Retroviridae family retrived from GeneBank (https://www.ncbi.nlm.nih.gov/genbank/), were aligned with ClustalW software (www.ebi.ac.uk/tools/msa/clustalw2/). The phylogenetic tree was generated by using the maximum likelihood procedure with MEGA 10.1 software (https://www.megasoftware.net).

## Data Availability

The datasets used and/or analysed during the current study are available from the corresponding author on reasonable request. All the sequences are registered on the NCBI database, and these are their links: Jaagsiekte sheep retrovirus, LTR, partial sequence (RO-24); https://www.ncbi.nlm.nih.gov/nuccore/MT809678.1 Exogenous Jaagsiekte sheep retrovirus, complete genome (type I) https://www.ncbi.nlm.nih.gov/nuccore/M80216; M80216 Exogenous Jaagsiekte sheep retrovirus (type II), https://www.ncbi.nlm.nih.gov/nuccore/AF105220; AF105220 *Ovis aries* endogenous virus Jaagsiekte sheep retrovirus, complete genome, https://www.ncbi.nlm.nih.gov/nuccore/dq838493.1; DQ838493 Ovine enzootic nasal tumor virus clone ENTVa LTR, complete sequence, https://www.ncbi.nlm.nih.gov/nuccore/KF199136; KF199136 Ovine enzootic nasal tumor virus clone ENTVb LTR, complete sequence, https://www.ncbi.nlm.nih.gov/nuccore/KF199137.1; KF199137 Ovine enzootic nasal tumor virus clone ENTVc LTR, complete sequence, https://www.ncbi.nlm.nih.gov/nuccore/KF199138; KF199138 Visna virus 3' LTR U3 region, https://www.ncbi.nlm.nih.gov/nuccore/M10131; M10131 Avian leukosis virus (RAV-2) circle junction (LTR), https://www.ncbi.nlm.nih.gov/nuccore/K03528; K03528 Equine infectious anemia virus clone Bulgan3 LTR LTR repeat region, https://www.ncbi.nlm.nih.gov/nuccore/MK579189; MK579189 Bovine leukemia virus strain 701 LTR, partial sequence, https://www.ncbi.nlm.nih.gov/nuccore/DQ287271; DQ287271 Simian Mason-Pfizer D-type retrovirus (MPMV/6A), complete genome, https://www.ncbi.nlm.nih.gov/nuccore/M12349; M12349 Mouse mammary tumor virus proviral LTR, U3 region, https://www.ncbi.nlm.nih.gov/nuccore/M14196; M14196 Squirrel monkey retrovirus 5' LTR, https://www.ncbi.nlm.nih.gov/nuccore/M13260; M13260 Simian retrovirus 2 isolate MfET1006, complete genome, https://www.ncbi.nlm.nih.gov/nuccore/KY235266; KY235266 Endogenous langur type D retrovirus PO-1-Lu gp70 gene, partial cds, https://www.ncbi.nlm.nih.gov/nuccore/AY282754; AY282754 Feline leukemia virus LTR, complete sequence, https://www.ncbi.nlm.nih.gov/nuccore/AY374189; AY374189

## Abbreviations

OPA: Ovine pulmonary adenocarcinoma
ExJSRV: Exogenous Jaagsiekte Sheep Retrovirus
ICC: Immunocytochemistry
IHC: Immunohistochemistry
MGs: Myxoid growths
JSRV-MA: Jaagsiekte Sheep Retrovirus-Matrix
MCK: Multi-cytokeratin
TTF: 1-thyroid transcription factor 1
SMA: Smooth muscle actin
alpha-SMA: alpha smooth muscle actin
Vim: Vimentin
UK: United Kingdom
LTR: Long terminal repeat
JSRV: Jaagsiekte Sheep Retrovirus
USA: United States of America
PCR: Polymerase chain reaction
DQP: Dia Quick Panoptic
H&E: Hematoxylin-eosin
AB-PAS: Alcian blue-periodic acid Shiff stain
DAB: 3, 3′-diaminobenzidine
TEM: Transmission electron microscopy
DNA: Deoxyribonucleic acid
UV: Ultraviolet
JSRV-RO: Jaagsiekte Sheep Retrovirus - ROmanian strain
N/C: Nuclear/cytoplasmic ratio
HPF: High power field
Bp: Base pair
RT: PCR-Reverse Transcription Polymerase Chain Reaction
nm: nanometer
μm: micrometer
